# Phenotypic and Genomic Characterization of ST133 Siderophore-Encoding Extensively Drug-Resistant Enterobacter hormaechei

**DOI:** 10.1128/aac.01737-22

**Published:** 2023-03-15

**Authors:** Yonglu Huang, Yuchen Wu, Chang Cai, Rong Zhang, Gongxiang Chen, Ning Dong

**Affiliations:** a Department of Clinical Laboratory, Second Affiliated Hospital of Zhejiang University, School of Medicine, Hangzhou, China; b China Australia Joint Laboratory for Animal Health Big Data Analytics, College of Animal Science and Technology, Zhejiang Agricultural and Forestry University, Hangzhou, China; c Department of Medical Microbiology, School of Biology and Basic Medical Science, Medical College of Soochow University, Suzhou, China; d Suzhou Key Laboratory of Pathogen Bioscience and Anti-infective Medicine, Soochow University, Suzhou, China

**Keywords:** *Enterobacter hormaechei*, ST133, *mcr-9*, *bla*_KPC-2_, increased virulence, siderophore, genomic characterization, extensive drug resistance, hypervirulence

## Abstract

We identified an ST133 extensively drug-resistant Enterobacter hormaechei, C210017, with increased virulence in the Galleria mellonella infection model. Genomic analysis suggested it carried antibiotic resistance genes *bla*_KPC-2_ and *mcr-9.1*, and genes *iutAiucABCD* and *iroBCDEN* encoding the virulence factor, siderophores. Comparative genomics of C210017 and the 178 ST133 *E. hormaechei* strains in the database suggested they all belonged to serotype O3 and most strains (77.5%) carried the IncHI2 superplasmids associated with the resistance, virulence, and adaptation of the host strain.

## INTRODUCTION

The genus Enterobacter is a member of the notorious ESKAPE pathogens which represent a global threat to human health (1). Enterobacter cloacae complex (ECC) accounted for 65% to 75% of Enterobacter infections in clinical settings (2, 3). ECC strains were frequently associated with a multidrug resistance (MDR) phenotype, and of particular concern is their resistance to the last-line antibiotics such as carbapenems and colistin (4). Enterobacter spp. generally use virulence factors typical of other Enterobacteriaceae which facilitate their survival in diverse environments (5). ECC is conventionally considered a low-virulence pathogen with only a few studies reported the characterization of virulent clones (6, 7). Here, as part of our routine resistance surveillance, we characterized an ST133 Enterobacter hormaechei strain that carried last-line antimicrobial resistance genes (ARGs, *mcr-9.1*, and *bla*_KPC-2_) and a cassette of virulence genes. Considering that ST133 could be an emerging high-risk ECC clone, we further studied the genomic and phylogenetic characteristics of all available ST133 genomes in the NCBI database.

**FIG 1:**
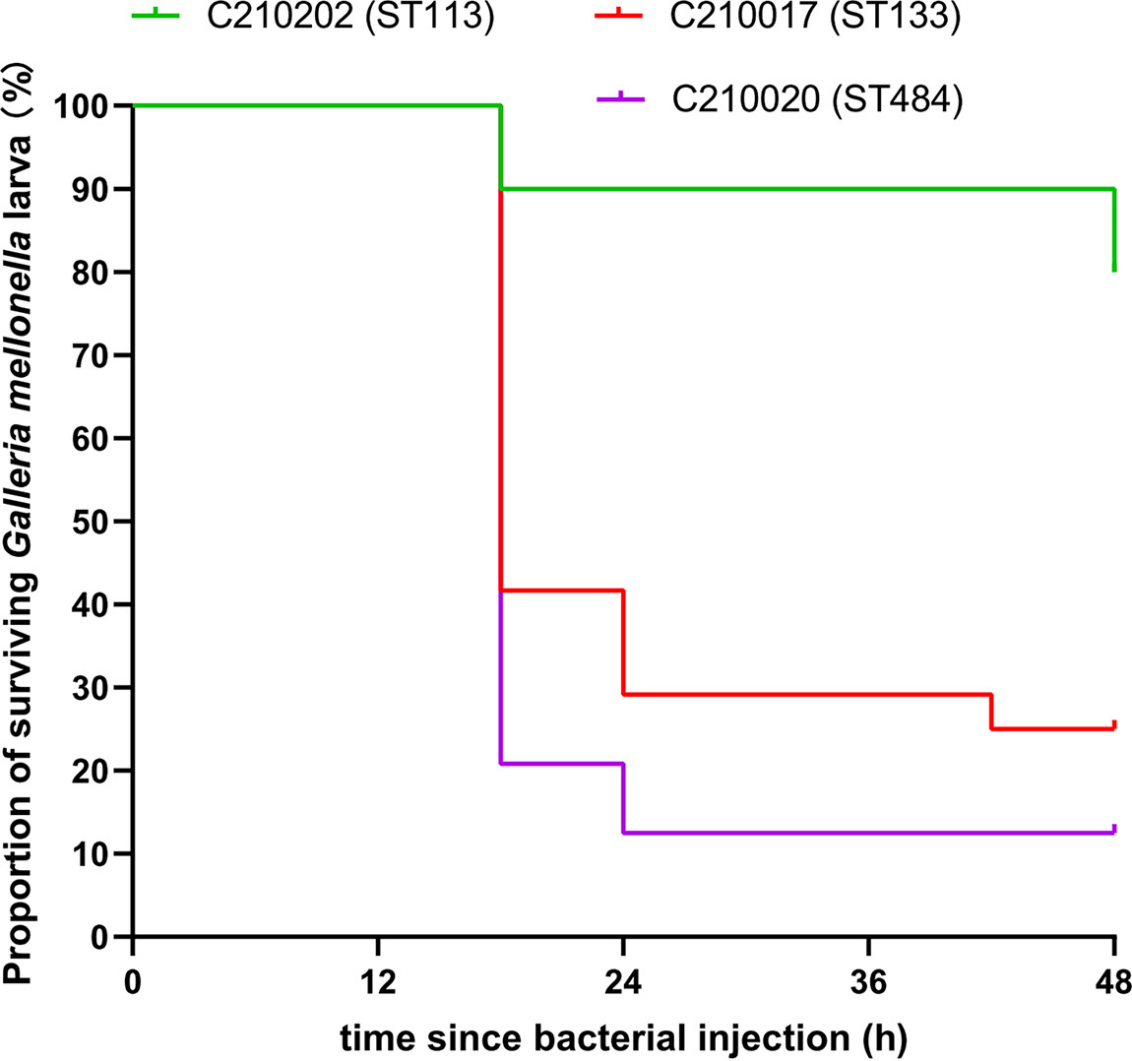
Virulence potential of the Enterobacter hormaechei strain C210017 in the G. mellonella infection model. Survival rates of G. mellonella (24 larvae in each group) infected with 1 × 10^6^ CFU of indicated isolates were shown by the Kaplan-Meier curve. The ST484 *E. hormaechei* strain C210020 was used as a control of hypervirulence and the ST113 *E. hormaechei* strain C210202 was used as the hypovirulence control strain.

A retrospective study led to the identification of an MCR-9- and KPC-2-producing *E. hormaechei* strain, C210017 isolated in 2020 from the fecal sample of a 54-year-old female patient in China (4). The genome of C210017 shared the average nucleotide identities of 98.76%, 97.49%, 97.11%, 95.89% (calculated with fastANI) to that of the type strains *E. hormaechei* subsp. *steigerwaltii* (GenBank accession: CP017179), *E. hormaechei* subsp. *oharae* (CP017180), *E. hormaechei* subsp. *xiangfangensis* (CP017183), *E. hormaechei* subsp. *hoffmannii* (CP017186), suggesting that C210017 belonged to *E. hormaechei* subsp. *steigerwaltii* (8). Antimicrobial susceptibility testing with the broth dilution method suggested that C210017 was resistant to carbapenems, tigecycline, ciprofloxacin, and amikacin. However, it remained susceptible to meropenem-vaborbactam, imipenem-relebactam, ceftazidime-avibactam, and colistin (Table 1). The virulence potential of C210017 was tested in the G. mellonella model (9). At the inoculum of 1 × 10^6^ CFU at 48 h after infection, survival rates of G. mellonella were 25.0% (95% CI = 10.2% to 43.1%) and 12.5% (95% CI = 3.1% to 28.7%) with *E*. *hormaechei* C210017 and the hypervirulence control strain *E*. *hormaechei* C210020, respectively. The survival rate recorded for the hypovirulence control strain *E*. *hormaechei* C210202 was 79.2% (95% CI = 55.1% to 92.0%) (Fig. 1). These data suggested that C210017 was simultaneously extensively drug resistant and highly virulent.

**TABLE 1:** MIC (mg/L) profiles of strains in this study^*a*^

Strain	IPM	MEM	ETP	CMZ	CAZ	CTX	TZP	CAV	FEP	PB	TGC	CIP	AK	ATM	MEM-VAB	IPM-REL
C210017	8	16	32	>128	128	>128	256/4	<0.5/4	>64	1	2	2	8	>128	1/8	<0.5/4
EC600-TC	4	4	16	4	32	16	256/4	<0.5/4	16	≤0.5	≤0.25	2	8	64	<0.5/8	<0.5/4
EC600	≤1	≤1	≤1	≤1	≤1	≤1	≤4	<0.5/4	≤1	≤0.5	≤0.25	≤0.25	≤2	≤1	<0.5/8	<0.5/4

a IPM, imipenem; MEM, meropenem; ETP, ertapenem; CMZ, cefmetazole; CAZ, ceftazidime; CTX, cefotaxime; TZP, piperacillin-tazobactam; CAV, ceftazidime-avibactam; FEP, cefepime; PB, polymyxin B; TGC, tigecycline; CIP, ciprofloxacin; AK, amikacin; ATM, aztreonam; MEM-VAB, meropenem-vaborbactam; IPM-REL, imipenem-relebactam.

Whole-genome sequencing was performed by both Illumina NovaSeq 6000 and Oxford nanopore MinION. The genome of strain C210017 was assembled into three complete circularized contigs using Unicycler 0.4.4 (10), including one chromosome and two plasmids pC210017_mcr and pC210017_KPC. The chromosome of C210017 was 4,938,556 bp, encoding 4737 ORFs with a G+C content of 55.4%. It carried ARGs *fosA* and *bla*_ACT-17_-like. C210017 belonged to sequence type ST133 and serotype O3. It carried multiple virulence-associated genes on its chromosome, including the *iutAiucABCD* and *iroBCDEN* gene clusters encoding the siderophores aerobactin and salmochelin, respectively, *flgGH* and *fliAGMQ* encoding flagella, *csgBA* encoding curli, and *hcp* encoding hemolysin-coregulated protein (11). TMost of these virulence genes were detected in >80% of the 9,919 ECC genomes from the NCBI database, but some genes were not commonly detected, including *iroBCDEN* carried by only 27% genomes and *hcp* in 66% genomes. Siderophores are important for bacteria, including ECC to scavenge iron for survival and for the establishment of infections (11). The virulence genes *iutAiucABCD*, *iroBCDEN* and *hcp* as well as other unidentified virulence factors could have contributed to the enhanced virulence of C210017 in the G. mellonella model.

**FIG 2:**
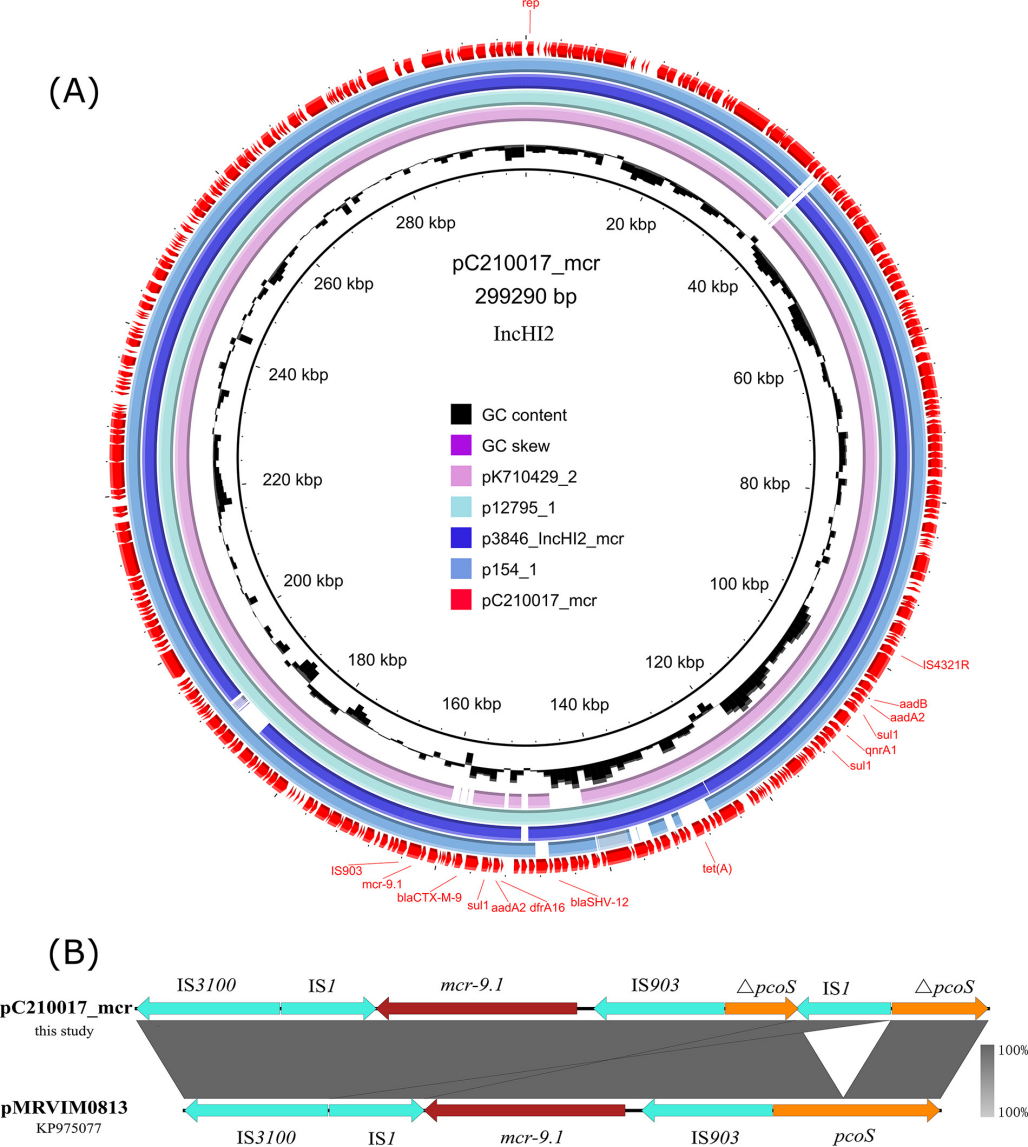
Genetic contents of plasmid pC210017_mcr in *E. hormaechei* strain C210017. (A) Map of plasmid pC210017_mcr. pC210017_mcr was aligned with plasmids with similar backbone in the NCBI database, including pK710429_2 (CP073658), p12795_1 (CP083854), p3846_IncHI2_mcr (CP052871), and p154_1 (CP038654). Antimicrobial resistance genes, plasmid replicons, and some mobile elements were labeled on the outermost circle. (B) Genetic context of *mcr-9.1* aligned to the homologous region in plasmid pMRVIM0813 (KP975077). Red, green, and yellow arrows indicate antimicrobial resistance genes, mobile elements, and other functional genes, respectively.

Plasmid pC210017_mcr was 299,290 bp with the G+C content of 47%. It was an IncHI2 plasmid encoding 337 ORFs. pC210017_mcr was highly identical to the IncHI2 “superplasmids” in diverse Enterobacteriaceae species such as Enterobacter sp., Klebsiella sp., *Citrobacter* sp., and Escherichia sp. with >99.9% identity and > 90% coverage. It carried ARGs, including *mcr-9.1*, *bla*_CTX-M-9_, *sul1* (three copies), *aadA2* (two copies), *dfrA16*, *bla*_SHV-12_, *tet*(A), *qnrA1*, and *aadB* all located on a ca. 60 Kb MDR region. This region was adjacent to insertion sequences IS*903* and IS*4321R* on each side, suggesting it could be acquired by genetic recombination mediated by insertion sequences (Fig. 2A). The genetic context of *mcr-9.1* was IS*3000*-IS*1*-*mcr-9.1*-IS*903*-△*pcoS*-IS*1*-△*pcoS*, similar to that in other IncHI2 “superplasmids” in Enterobacteriaceae (Fig. 2B). Conjugation with the filter-mating method failed to transfer pC210017_mcr to the rifampicin-resistant E. coli EC600.

**FIG 3:**
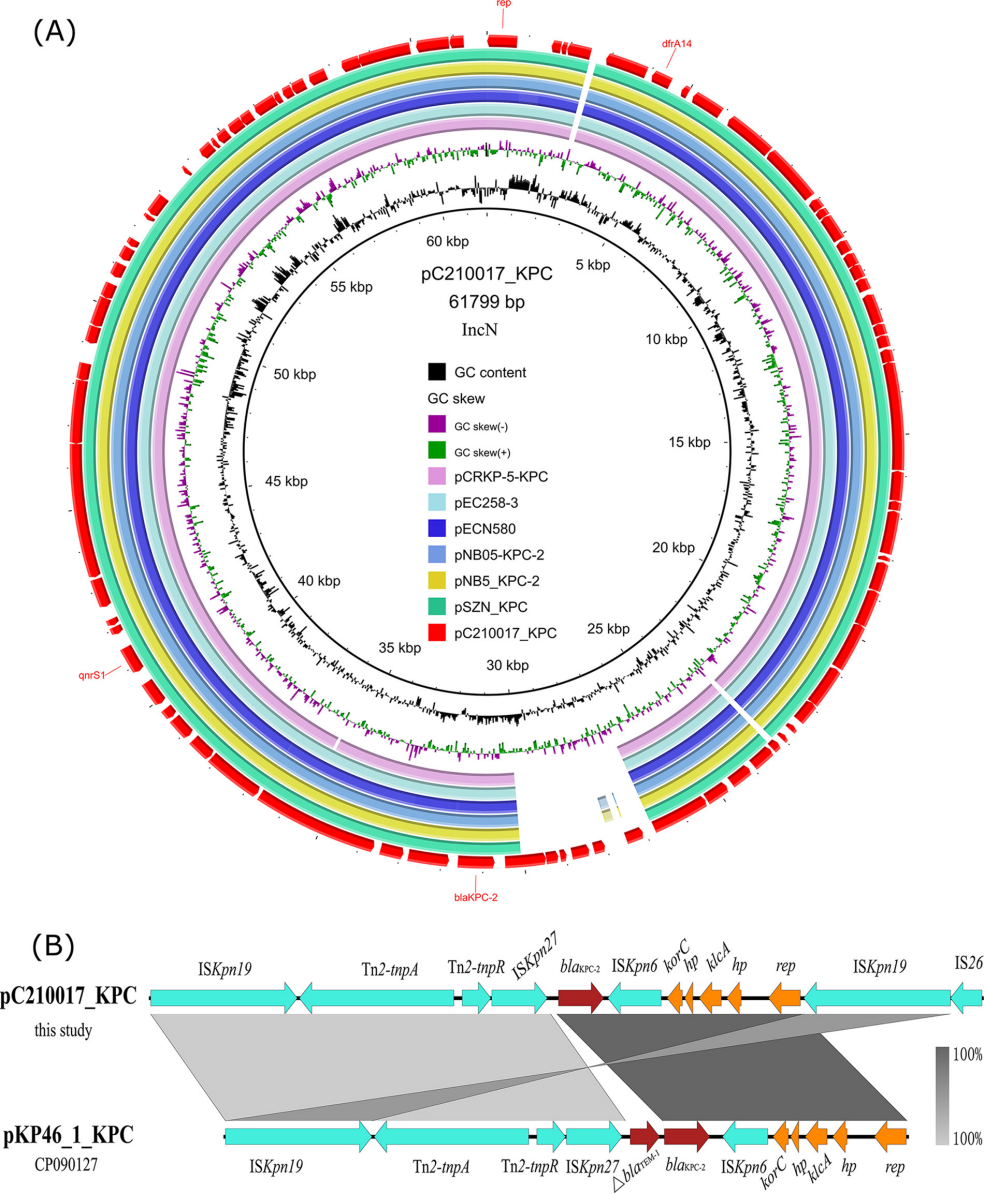
Genetic contents of plasmid pC210017_KPC in *E. hormaechei* strain C210017. (A) Map of plasmid pC210017_KPC. pC210017_KPC was aligned with plasmids with similar backbone in the NCBI database, including pCRKP-5-KPC (KX928751), pEC258-3 (CP097098), pECN580 (KF914891), pNB05-KPC-2 (CP091849), pNB5_KPC-2 (CP092655), and pSZN_KPC (MH917123). Antimicrobial resistance genes, plasmid replicons and some mobile elements were labeled on the outermost circle. (B) Genetic context of *bla*_KPC-2_ aligned to the homologous region in plasmid pKP46_1_KPC (CP090127). Red, green, and yellow arrows indicate antimicrobial resistance genes, mobile elements and other functional genes, respectively.

Plasmid pC210017_KPC was 61,799 bp in length with a G+C content of 51.6%. It was an IncN plasmid encoding 83 ORFs. pC210017_KPC was 99.98% identical to plasmid pSZN_KPC (MH917123) from a K. pneumoniae strain at 94% coverage. It also exhibited >90% identity to plasmids pCRKP-5-KPC (KX928751), pEC258-3 (CP097098), pECN580 (KF914891), pNB05-KPC-2 (CP091849), and pNB5 KPC-2 (CP092655). pC210017_KPC carried ARGs *bla*_KPC-2_, *qnrS1*, and *dfrA14* (Fig. 3A). The genetic context of *bla*_KPC-2_ was IS*Kpn19*-Tn*2*-IS*Kpn27*-*bla*_KPC-2_-IS*Kpn6*-*korC*-*hp*-*klcA*-*hp*-*rep*-IS*Kpn19*-IS*26* (Fig. 3B). pC210017_KPC could be transferred to E. coli EC600 via conjugation at the frequency of 1 × 10^−3^ (Table 1).

**FIG 4:**
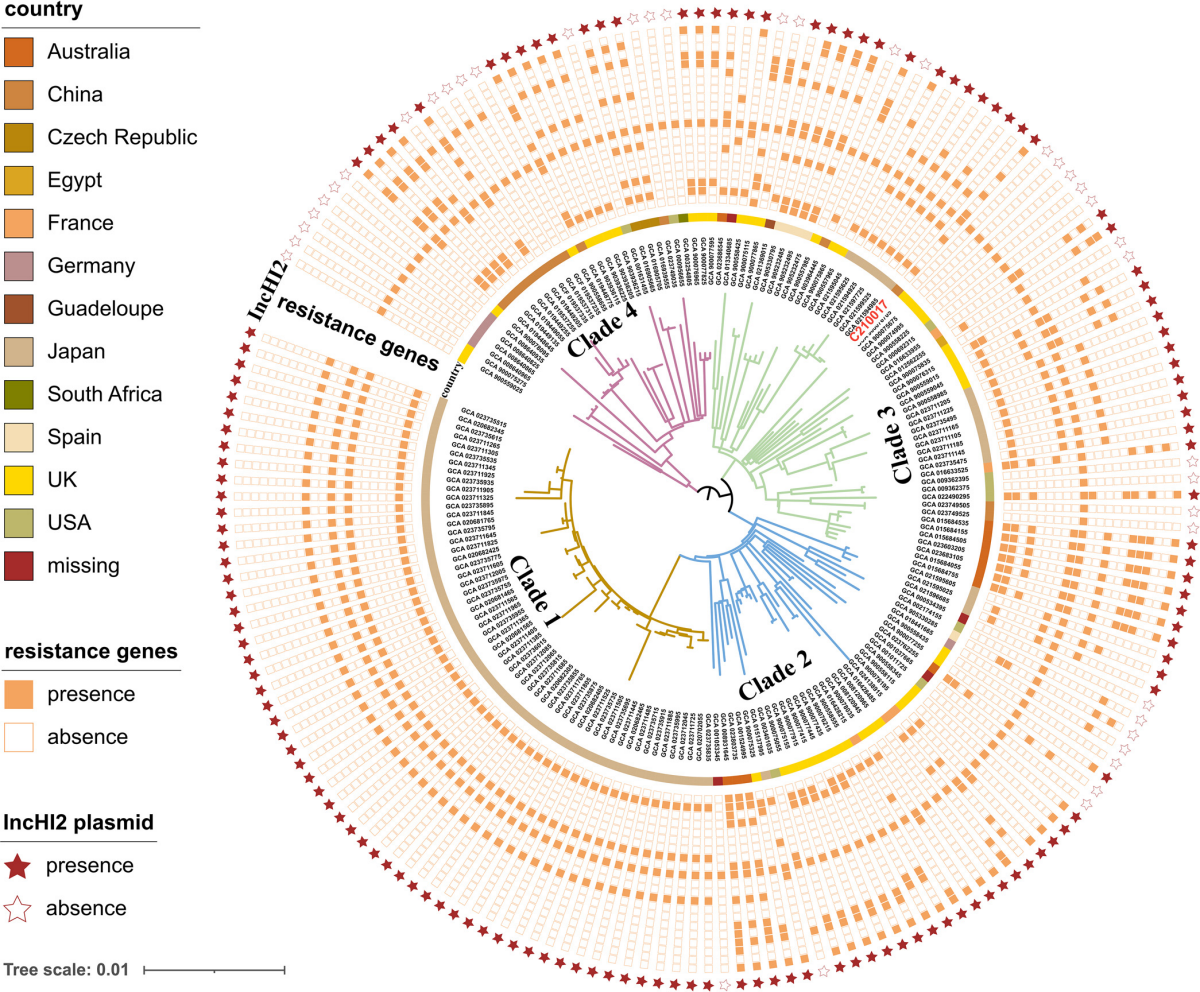
Phylogenetic analysis of ST133 *E. hormaechei*. Branch lengths were ignored to optimize the tree. The four clades defined by fastbaps were labeled with different colors of branches. The circles bordering the phylogenetic tree include source of isolation, carriage of antimicrobial resistance genes and the presence of IncHI2 plasmids among these ST133 *E. hormaechei*. the Resistance genes were *bla*_ACT_, *bla*_CTX_, *bla*_DHA_, *bla*_IMP_, *bla*_KPC_, *bla*_NDM_, *bla*_OXA_, *bla*_SHV-12_, *bla*_TEM-1B_, *bla*_VEB-3_, *bla*_VIM-1_, and *mcr-9* from the innermost to the outermost circle.

A total of 178 ST133 Enterobacter spp. were detected in 9,919 ECC genomes retrieved from the NCBI nt/nr database on July 23, 2022. All ST133 strains were classified as *E*. *hormaechei* subsp. *steigerwaltii* and all belonged to serotype O3. They were isolated from different countries across the world (Table S1). Four major phylogroups were defined with fastBAPS based on the SNP of the 179 ST133 *E*. *hormaechei* strains, including C210017, and each group included 57, 36, 54, and 32 strains (Table S2) (12). C210017 belonged to clades 3 which also included strains from European, American, Australian, and other Asian countries, suggesting this virulent and hyper-resistant clone could have undergone global dissemination. All 178 ST133 *E*. *hormaechei* strains harbored ARGs, including 102 strains carrying at least one carbapenemase gene (Fig. S1). Co-occurrence of *mcr-9* and carbapenemase genes were observed in 29/178 strains. IncHI2 plasmids which were reported to be frequently associated with resistance genes such as *mcr-9* in Enterobacter sp. was detected in 138/178 (77.5%) ST133 genomes (Fig. 4). Virulence genes, including *iutAiucABCD*, *iroBCDEN*, *flgGH*, *fliAGMQ*, *csgBA*, and *hcp* carried by strain C210017 were all detected in all ST133 strains in the NCBI database. These data suggested that ST133 *E. hormaechei* could be an important reservoir for virulent Enterobacter sp. (Table S3).

ECC is conventionally considered a low-virulence pathogen before the recent report of hypervirulent *E. bugandensis* which was responsible for fatal bacteremia of neonates in a neonatal intensive care unit in France (6). Thereafter, Xu et al. reported an epidemic carbapenem-resistant hypervirulent clone (ST133) of E. hormaechei showing high invasiveness (7). However, the genomic characteristics and transferability of ST133 ECC were not comprehensively studied previously. Results in our study boosted our understanding of this emerging pathogen. The limitation of this study included (i) the patient's clinical manifestations and outcome could not be obtained, and (ii) the virulence potential of only one ST133 ECC was investigated with the G. mellonella infection model. The virulence phenotypes of more Enterobacter strains remained to be investigated with more infection models.

### Conclusions.

In summary, we reported the emergence of an ST133 extensively drug resistant and virulent *E. hormaechei* carrying *mcr-9.1*, *bla*_KPC-2_ and siderophore-encoding genes. The 178 ST133 *E. hormaechei* genomes in the NCBI database were also characterized, all of which carried virulence factors similar to that of strain C210017 and ARGs. They were clustered into four major clades. The IncHI2 “superplasmids” which could be associated with the resistance, virulence, and adaptation of Enterobacter strains were frequently detected among the ST133 genomes. ST133 *E. hormaechei* has spread around the world. Our results highlighted the emergence of a potential high-risk clone, which could exacerbate the lack of clinical choices for infection therapy.

### Ethical approval.

The study was approved by the Ethics Committee of Second Affiliated Hospital, Zhejiang University School of Medicine. The subjects gave written informed consent in accordance with the Declaration of Helsinki.

### Data availability.

The genome sequence of E. hormaechei strain C210017 has been deposited in the GenBank database under the BioSample accession number: SAMN28416099.
