# Supplementary material for: Phenotypic and Genomic Characterization of ST133 Siderophore-Encoding Extensively Drug-Resistant Enterobacter hormaechei
Source: Antimicrob Agents Chemother. 2023 Mar 15;67(4):e01737-22. doi: 10.1128/aac.01737-22 (PMC10112160; doi:10.1128/aac.01737-22)
Supplement: Supplemental file 2 — Supplemental material. Download aac.01737-22-s0002.pdf, PDF file, 2.0 MB [file aac.01737-22-s0002.pdf]

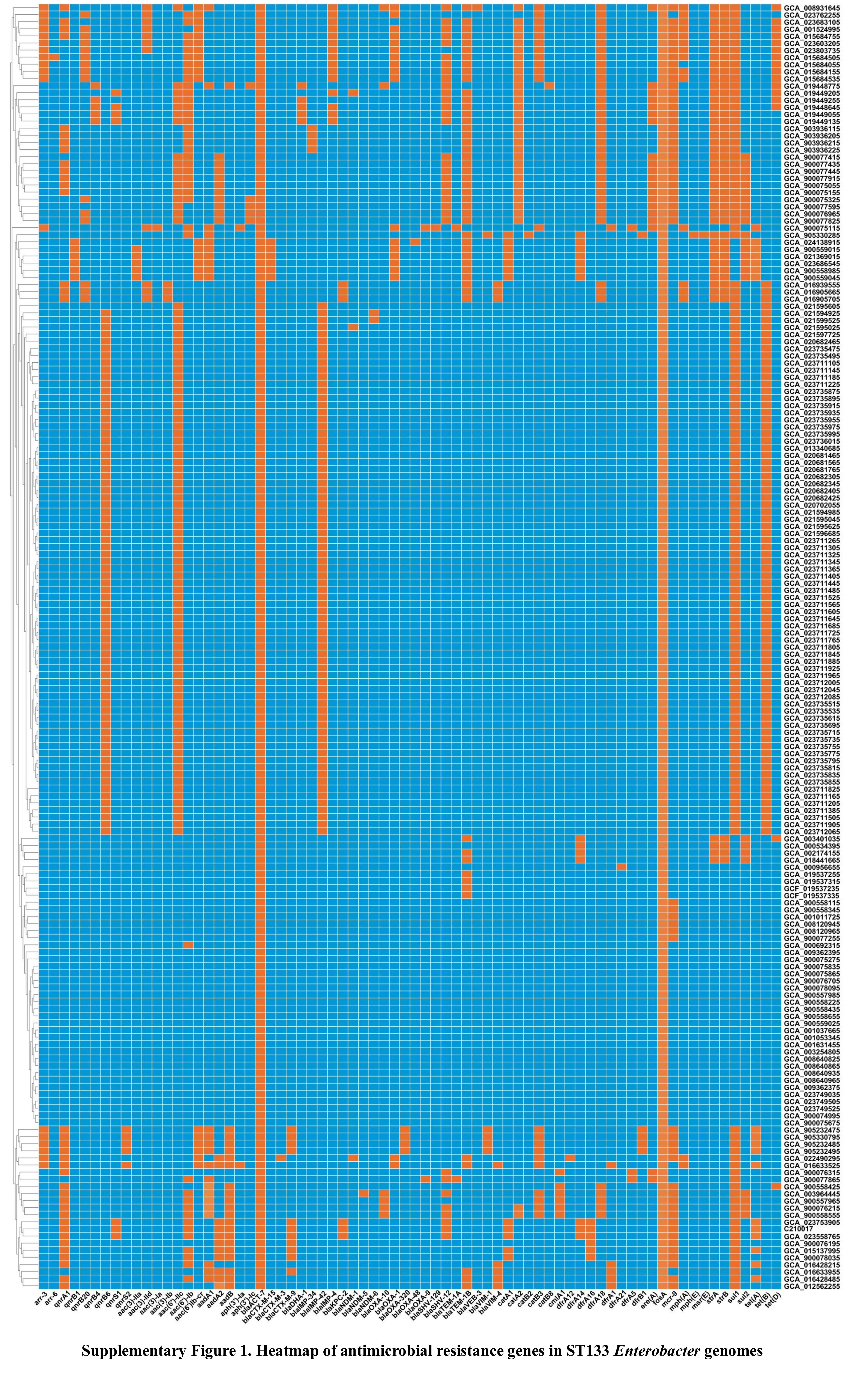

Supplementary Figure 1. Heatmap of antimicrobial resistance genes in ST133 *Enterobacter* genomes

Supplementary Table 1. Information of ST133 *Enterobacter* from the NCBI database

Accession	Country	Serotype
GCA_000534395	missing	O3
GCA_000692315	USA	O3
GCA_000956655	USA	O3
GCA_001011725	USA	O3
GCA_001037665	missing	O3
GCA_001053345	missing	O3
GCA_001524995	Australia	O3
GCA_001631455	USA	O3
GCA_002174155	USA	O3
GCA_003254805	South Africa	O3
GCA_003401035	USA	O3
GCA_003964445	China	O3
GCA_008120945	UK	O3
GCA_008120965	UK	O3
GCA_008640825	Germany	O3
GCA_008640865	Germany	O3
GCA_008640935	Germany	O3
GCA_008640965	Germany	O3
GCA_008931645	Australia	O3
GCA_009362375	USA	O3
GCA_009362395	USA	O3
GCA_012562255	Egypt	O3
GCA_013340685	missing	O3
GCA_015137995	Japan	O3
GCA_015684055	Australia	O3
GCA_015684155	Australia	O3
GCA_015684505	Australia	O3
GCA_015684535	Australia	O3
GCA_015684755	Australia	O3
GCA_016428215	France	O3
GCA_016428485	France	O3
GCA_016633525	France	O3
GCA_016633955	France	O3
GCA_016905665	Czech Republic	O3
GCA_016905705	Czech Republic	O3
GCA_016939555	Czech Republic	O3
GCA_018441665	Germany	O3

GCA_019448645	China	O3
GCA_019448775	China	O3
GCA_019449055	China	O3
GCA_019449135	China	O3
GCA_019449205	China	O3
GCA_019449255	China	O3
GCA_019537255	China	O3
GCA_019537315	China	O3
GCA_020681465	Japan	O3
GCA_020681565	Japan	O3
GCA_020681765	Japan	O3
GCA_020682305	Japan	O3
GCA_020682345	Japan	O3
GCA_020682405	Japan	O3
GCA_020682425	Japan	O3
GCA_020682465	Japan	O3
GCA_020702055	Japan	O3
GCA_021369015	Guadeloupe	O3
GCA_021594925	Japan	O3
GCA_021594985	Japan	O3
GCA_021595025	Japan	O3
GCA_021595045	Japan	O3
GCA_021595605	Japan	O3
GCA_021595625	Japan	O3
GCA_021596685	Japan	O3
GCA_021597725	Japan	O3
GCA_021599525	Japan	O3
GCA_022490295	USA	O3
GCA_023603205	Australia	O3
GCA_023683105	Australia	O3
GCA_023686545	Australia	O3
GCA_023711105	Japan	O3
GCA_023711145	Japan	O3
GCA_023711165	Japan	O3
GCA_023711185	Japan	O3
GCA_023711205	Japan	O3
GCA_023711225	Japan	O3
GCA_023711265	Japan	O3
GCA_023711305	Japan	O3

GCA_023711325	Japan	O3
GCA_023711345	Japan	O3
GCA_023711365	Japan	O3
GCA_023711385	Japan	O3
GCA_023711405	Japan	O3
GCA_023711445	Japan	O3
GCA_023711485	Japan	O3
GCA_023711505	Japan	O3
GCA_023711525	Japan	O3
GCA_023711565	Japan	O3
GCA_023711605	Japan	O3
GCA_023711645	Japan	O3
GCA_023711685	Japan	O3
GCA_023711725	Japan	O3
GCA_023711765	Japan	O3
GCA_023711805	Japan	O3
GCA_023711825	Japan	O3
GCA_023711845	Japan	O3
GCA_023711885	Japan	O3
GCA_023711905	Japan	O3
GCA_023711925	Japan	O3
GCA_023711965	Japan	O3
GCA_023712005	Japan	O3
GCA_023712045	Japan	O3
GCA_023712065	Japan	O3
GCA_023712085	Japan	O3
GCA_023735475	Japan	O3
GCA_023735495	Japan	O3
GCA_023735515	Japan	O3
GCA_023735535	Japan	O3
GCA_023735615	Japan	O3
GCA_023735695	Japan	O3
GCA_023735715	Japan	O3
GCA_023735735	Japan	O3
GCA_023735755	Japan	O3
GCA_023735775	Japan	O3
GCA_023735795	Japan	O3
GCA_023735815	Japan	O3
GCA_023735835	Japan	O3

GCA_023735855	Japan	O3
GCA_023735875	Japan	O3
GCA_023735895	Japan	O3
GCA_023735915	Japan	O3
GCA_023735935	Japan	O3
GCA_023735955	Japan	O3
GCA_023735975	Japan	O3
GCA_023735995	Japan	O3
GCA_023736015	Japan	O3
GCA_023749035	China	O3
GCA_023749505	China	O3
GCA_023749525	China	O3
GCA_023762255	Australia	O3
GCA_023803735	Australia	O3
GCA_024138915	France	O3
GCA_900074995	UK	O3
GCA_900075055	UK	O3
GCA_900075115	UK	O3
GCA_900075155	UK	O3
GCA_900075275	UK	O3
GCA_900075325	UK	O3
GCA_900075675	UK	O3
GCA_900075835	UK	O3
GCA_900075865	UK	O3
GCA_900076195	UK	O3
GCA_900076215	UK	O3
GCA_900076315	UK	O3
GCA_900076705	UK	O3
GCA_900076965	UK	O3
GCA_900077255	UK	O3
GCA_900077415	UK	O3
GCA_900077435	UK	O3
GCA_900077445	UK	O3
GCA_900077595	UK	O3
GCA_900077825	UK	O3
GCA_900077865	UK	O3
GCA_900077915	UK	O3
GCA_900078035	UK	O3
GCA_900078095	UK	O3

GCA_900557965	UK	O3
GCA_900557985	UK	O3
GCA_900558115	UK	O3
GCA_900558225	UK	O3
GCA_900558345	UK	O3
GCA_900558425	UK	O3
GCA_900558435	UK	O3
GCA_900558555	UK	O3
GCA_900558655	UK	O3
GCA_900558985	UK	O3
GCA_900559015	UK	O3
GCA_900559025	UK	O3
GCA_900559045	UK	O3
GCA_903936115	UK	O3
GCA_903936205	UK	O3
GCA_903936215	UK	O3
GCA_903936225	UK	O3
GCA_905232475	spain	O3
GCA_905232485	spain	O3
GCA_905232495	spain	O3
GCA_905330285	spain	O3
GCA_905330795	spain	O3
GCF_019537235	China	O3
GCF_019537335	China	O3

Supplementary Table 3 Carriage of virulence-associated genes in ST133 *Enterobacter* genomes

	cheB	*cheY*	*fepD*	*flgG*	*flgH*	*fliA*	*fliG*	*fliM*	*fliQ*	*hcp*	*iroB*	*iroC*	*iroD*	*iroE*	*iroN*	*motB*	*ompA*	*tcyJ*
C210017	82.156	83.376	80.373	84.419	84.531	86.092	87.1	84.623	81.203	85.976	90.135	89.381	87.805	87.214	91.736	81.043	85.523	83.333
GCA_000534395	82.156	83.376	80.373	84.419	84.531	86.092	87.1	84.623	81.203	85.976	90.135	89.381	87.805	87.214	91.736	81.043	85.523	83.333
GCA_000692315	82.156	83.376	80.373	84.419	84.531	86.092	87.1	84.623	81.203	85.976	90.135	89.381	87.805	87.214	91.736	81.043	85.523	83.333
GCA_000956655	82.156	83.376	80.373	84.419	84.531	86.092	87.1	84.623	81.203	85.976	90.135	89.381	87.805	87.214	91.736	81.043	85.523	83.333
GCA_001011725	82.156	83.376	80.373	84.419	84.531	86.092	87.1	84.623	81.203	85.976	90.135	89.381	87.805	87.214	91.736	81.043	85.523	83.333
GCA_001037665	82.156	83.376	80.373	84.419	84.531	86.092	87.1	84.623	81.203	85.976	90.135	89.381	87.805	87.214	91.736	81.043	85.523	83.333
GCA_001053345	82.156	83.376	80.373	84.419	84.531	86.092	87.1	84.623	81.203	85.976	90.135	89.381	87.805	87.214	91.736	81.043	85.523	83.333
GCA_001524995	82.156	83.376	80.373	84.419	84.531	86.092	87.1	84.623	81.203	85.976	90.135	89.381	87.805	87.11	91.736	81.043	85.523	83.333
GCA_001631455	82.156	83.376	80.373	84.419	84.531	86.092	87.1	84.623	81.203	85.976	90.135	89.381	87.805	87.214	91.736	81.043	85.523	83.333
GCA_002174155	82.156	83.376	80.373	84.419	84.531	86.092	87.1	84.623	81.203	85.976	90.135	89.381	87.805	87.214	91.736	81.043	85.523	83.333
GCA_003254805	82.156	83.376	80.373	84.419	84.531	86.092	87.1	84.623	81.203	85.976	90.135	89.381	87.805	87.214	91.736	81.043	85.523	83.333
GCA_003401035	82.156	83.376	80.373	84.419	84.531	86.092	87.1	84.623	81.203	85.976	90.135	89.381	87.805	87.214	91.736	81.043	85.523	83.333
GCA_003964445	82.156	83.376	80.373	84.419	84.531	86.092	87.1	84.623	81.203	85.976	90.045	89.381	87.805	87.214	91.736	81.043	85.523	83.333
GCA_008120945	82.156	83.376	80.373	84.419	84.531	86.092	87.1	84.623	81.203	85.976	90.135	89.381	87.805	87.214	91.736	81.043	85.523	83.333
GCA_008120965	82.156	83.376	80.373	84.419	84.531	86.092	87.1	84.623	81.203	85.976	90.135	89.381	87.805	87.214	91.736	81.043	85.523	83.333
GCA_008640825	82.156	83.376	80.373	84.419	84.531	86.092	87.1	84.623	81.203	85.976	90.135	89.381	87.805	87.214	91.736	81.043	85.523	83.333
GCA_008640865	82.156	83.376	80.373	84.419	84.531	86.092	87.1	84.623	81.203	85.976	90.135	89.381	87.805	87.214	91.736	81.043	85.523	83.333
GCA_008640935	82.156	83.376	80.373	84.419	84.531	86.092	87.1	84.623	81.203	85.976	90.135	89.381	87.805	87.214	91.736	81.043	85.523	83.333
GCA_008640965	82.156	83.376	80.373	84.419	84.531	86.092	87.1	84.623	81.203	85.976	90.135	89.381	87.805	87.214	91.736	81.043	85.523	83.333
GCA_008931645	82.156	83.376	80.373	84.419	84.531	85.953	87.1	84.623	81.203	85.976	90.135	89.354	87.805	87.11	91.736	81.043	85.523	83.333
GCA_009362375	82.156	83.376	80.373	84.419	84.531	86.092	87.1	84.623	81.203	85.976	90.135	89.381	87.805	87.214	91.736	81.043	85.523	83.333
GCA_009362395	82.156	83.376	80.373	84.419	84.531	86.092	87.1	84.623	81.203	85.976	90.135	89.381	87.805	87.214	91.736	81.043	85.523	83.333
GCA_012562255	82.156	83.376	80.373	84.419	84.531	86.092	87.1	84.623	81.203	85.976	90.135	89.381	87.805	87.214	91.736	81.043	85.523	83.333
GCA_013340685	82.156	83.376	80.373	84.419	84.531	86.092	87.1	84.623	81.203	85.976	90.135	89.381	87.805	87.214	91.736	81.043	85.523	83.333
GCA_015137995	82.156	83.376	80.373	84.419	84.531	86.092	87.1	84.623	81.203	85.976	90.135	89.354	87.724	87.11	91.736	81.043	85.523	83.333
GCA_015684055	82.156	83.376	80.373	84.419	84.531	86.092	87.1	84.623	81.203	85.976	90.135	89.381	87.805	87.214	91.736	81.043	85.523	83.333
GCA_015684155	82.156	83.376	80.373	84.439	84.531	86.092	87.1	84.623	81.203	85.976	90.135	89.381	87.805	87.214	91.736	81.043	85.523	83.333
GCA_015684505	82.156	83.376	80.373	84.439	84.531	86.092	87.1	84.623	81.203	85.976	90.135	89.381	87.805	87.214	91.736	81.043	85.523	83.333
GCA_015684535	82.156	83.376	80.373	84.439	84.531	86.092	87.1	84.623	81.203	85.976	90.135	89.381	87.805	87.418	91.736	81.043	85.523	83.333
GCA_015684755	82.156	83.376	80.373	84.419	84.531	86.092	87.1	84.623	81.203	85.976	90.135	89.381	87.805	87.214	91.736	81.043	85.523	83.333
GCA_016428215	82.156	83.376	80.373	84.419	84.531	86.092	87.1	84.623	81.203	85.976	90.135	89.381	87.805	87.214	91.736	81.043	85.523	83.333
GCA_016428485	82.156	83.376	80.373	84.419	84.531	86.092	87.1	84.623	81.203	85.976	90.135	89.381	87.805	87.214	91.736	81.043	85.523	83.333
GCA_016633525	82.156	83.376	0	84.419	84.531	86.092	87.1	84.623	81.203	85.976	90.135	89.354	87.805	87.214	91.736	81.043	85.523	83.333
GCA_016633955	82.156	83.376	80.373	84.419	84.531	86.092	87.1	84.623	81.203	85.976	90.135	89.381	87.805	87.214	91.736	81.043	85.523	83.333
GCA_016905665	82.156	83.376	80.373	84.419	84.531	86.092	87.1	84.524	81.203	85.976	90.135	89.381	87.805	87.214	91.736	81.043	85.523	83.333
GCA_016905705	82.156	83.376	80.373	84.419	84.531	86.092	87.1	84.524	81.203	85.976	90.135	89.381	87.805	87.214	91.736	81.043	85.523	83.333
GCA_016939555	82.156	83.376	80.373	84.419	84.531	86.092	87.1	84.524	81.203	85.976	90.135	89.381	87.805	87.214	91.736	81.043	85.523	83.333
GCA_018441665	82.156	83.376	80.373	84.419	84.531	86.092	87.1	84.623	81.203	85.976	90.135	89.381	87.805	87.214	91.736	81.043	85.523	83.333
GCA_019448645	82.156	83.376	80.373	84.419	84.531	86.092	87.1	84.623	81.203	85.976	90.135	89.381	87.805	87.214	91.736	81.043	85.523	83.333
GCA_019448775	82.156	83.376	80.373	84.419	84.531	86.092	87.1	84.623	81.203	85.976	90.135	89.381	87.805	87.214	91.736	81.043	85.523	83.333
GCA_019449055	82.156	83.376	80.373	84.419	84.531	86.092	87.1	84.623	81.203	85.976	90.135	89.381	87.805	87.214	91.736	81.043	85.523	83.333
GCA_019449135	82.156	83.376	80.373	84.419	84.531	86.092	87.1	84.623	81.203	85.976	90.135	89.381	87.805	87.214	91.736	81.043	85.523	83.333
GCA_019449205	82.156	83.376	80.373	84.419	84.531	86.092	87.1	84.623	81.203	85.976	90.135	89.381	87.805	87.214	91.736	81.043	85.523	83.333
GCA_019449255	82.156	83.376	80.373	84.419	84.531	86.092	87.1	84.623	81.203	85.976	90.135	89.381	87.805	87.214	91.736	81.043	85.523	83.333
GCA_019537255	82.156	83.376	80.373	84.419	84.531	86.092	87.1	84.623	81.203	85.976	90.135	89.381	87.805	87.214	91.736	81.043	85.523	83.333
GCA_019537315	82.156	83.376	80.373	84.419	84.531	86.092	87.1	84.623	81.203	85.976	90.135	89.381	87.805	87.214	91.736	81.043	85.523	83.333
GCA_020681465	82.156	83.376	80.373	84.419	84.531	86.092	87.1	84.623	81.203	85.976	90.135	89.381	87.805	87.214	91.69	81.043	85.523	83.333
GCA_020681565	82.156	83.376	80.373	84.419	84.531	86.092	87.1	84.623	81.203	85.976	90.135	89.381	87.805	87.214	91.69	81.043	85.523	83.333
GCA_020681765	82.156	83.376	80.373	84.419	84.531	86.092	87.1	84.623	81.203	85.976	90.135	89.381	87.805	87.214	91.69	81.043	85.523	83.333
GCA_020682305	82.156	83.376	80.373	84.419	84.531	86.092	87.1	84.623	81.203	85.976	90.135	89.381	87.805	87.214	91.69	81.043	85.523	83.333
GCA_020682345	82.156	83.376	80.373	84.419	84.531	86.092	87.1	84.623	81.203	85.976	90.135	89.381	87.805	87.214	91.69	81.043	85.523	83.333
GCA_020682405	82.156	83.12	80.373	84.419	84.531	86.092	87.1	84.623	81.203	85.976	90.135	89.381	87.805	87.11	91.69	81.043	85.523	83.333
GCA_020682425	82.156	83.376	80.373	84.419	84.531	86.092	87.1	84.623	81.203	85.976	90.135	89.381	87.805	87.214	91.69	81.043	85.523	83.333
GCA_020682465	82.156	83.376	80.373	84.419	84.531	86.092	87.1	84.623	81.203	85.976	90.135	89.381	87.805	87.214	91.69	81.043	85.523	83.333
GCA_020702055	82.156	83.376	80.373	84.419	84.531	86.092	87.1	84.623	81.203	85.976	90.135	89.381	87.805	87.214	91.69	81.043	85.523	83.333
GCA_021369015	82.156	83.12	80.373	84.419	84.531	86.092	87.1	84.623	81.203	85.976	90.135	89.381	87.805	87.214	91.736	80.916	85.523	83.333
GCA_021594925	82.156	83.376	80.373	84.419	84.531	86.092	87.1	84.623	81.203	85.976	90.135	89.381	87.805	87.214	91.736	81.043	85.523	83.333
GCA_021594985	82.156	83.376	80.373	84.419	84.531	86.092	87.1	84.623	81.203	85.976	90.135	89.381	87.805	87.214	91.736	81.043	85.523	83.333
GCA_021595025	82.156	83.376	80.373	84.419	84.531	86.092	87	84.425	81.203	85.976	90.135	89.381	87.805	87.214	91.736	81.043	85.523	83.333
GCA_021595045	82.156	83.376	80.373	84.419	84.531	86.092	87.1	84.623	81.203	85.976	90.135	89.381	87.805	87.214	91.736	81.043	85.523	83.333
GCA_021595605	82.156	83.376	80.373	84.419	84.531	86.092	87.1	84.524	81.203	85.976	90.135	89.381	87.805	87.214	91.736	81.043	85.523	83.333
GCA_021595625	82.156	83.376	80.373	84.419	84.531	86.092	87.1	84.623	81.203	85.976	90.135	89.381	87.805	87.214	91.736	81.043	85.523	83.333
GCA_021596685	82.156	83.376	80.373	84.419	84.531	86.092	87.1	84.524	81.203	85.976	90.135	89.381	87.805	87.214	91.736	81.043	85.523	83.333
GCA_021597725	82.156	83.376	80.373	84.419	84.531	86.092	87.1	84.623	81.203	85.976	90.135	89.381	87.805	87.214	91.736	81.043	85.523	83.333
GCA_021599525	82.156	83.376	80.373	84.419	84.531	86.092	87.1	84.623	81.203	85.976	90.135	89.381	87.805	87.214	91.736	81.043	85.523	83.333
GCA_022490295	82.156	83.376	80.373	84.419	84.531	86.092	87.1	84.623	81.203	85.976	90.135	89.381	87.724	87.214	91.736	81.043	85.523	83.333
GCA_023558765	82.156	83.376	80.373	84.419	84.531	86.092	87.1	84.623	81.203	85.976	90.135	89.381	87.805	87.214	91.736	81.043	85.523	83.333
GCA_023603205	82.156	83.376	80.373	84.419	84.531	86.092	87.1	84.623	81.203	85.976	90.135	89.381	87.805	87.214	91.736	81.043	85.523	83.333
GCA_023683105	82.156	83.376	80.373	84.439	84.531	86.092	87.1	84.623	81.203	85.976	90.135	89.381	87.805	87.214	91.736	81.043	85.523	83.333
GCA_023686545	82.156	83.376	80.373	84.419	84.531	86.092	87.1	84.623	81.203	85.976	90.135	89.381	87.805	87.214	91.736	81.043	85.523	83.333
GCA_023711105	82.156	83.376	80.373	84.419	84.531	86.092	87.1	84.623	81.203	85.976	90.135	89.381	87.805	87.214	91.736	81.043	85.523	83.333
GCA_023711145	82.156	83.376	80.373	84.419	84.531	86.092	87.1	84.623	81.203	85.976	90.135	89.381	87.805	87.214	91.736	81.043	85.523	83.333
GCA_023711165	82.156	83.376	80.373	84.419	84.531	86.092	87.1	84.623	81.203	85.976	90.135	89.354	87.724	87.214	91.736	81.17	85.523	83.333
GCA_023711185	82.156	83.376	80.373	84.419	84.531	86.092	87.1	84.623	81.203	85.976	90.135	89.381	87.805	87.214	91.736	81.043	85.523	83.333

GCA_023711205	82.156	83.376	80.373	84.419	84.531	86.092	87.1	84.623	81.203	85.976	90.135	89.381	87.642	87.214	91.736	81.17	85.523	83.333
GCA_023711225	82.156	83.376	80.373	84.419	84.531	86.092	87.1	84.623	81.203	85.976	90.135	89.381	87.805	87.214	91.736	81.043	85.523	83.333
GCA_023711265	82.156	83.376	80.373	84.419	84.531	86.092	87.1	84.623	81.203	85.976	90.135	89.381	87.805	87.214	91.69	81.043	85.523	83.333
GCA_023711305	82.156	83.376	80.373	84.419	84.531	86.092	87.1	84.623	81.203	85.976	90.135	89.381	87.805	87.214	91.69	81.043	85.523	83.333
GCA_023711325	82.156	83.376	80.461	84.419	84.531	86.092	87.1	84.623	81.203	85.976	90.135	89.354	87.642	87.214	91.69	81.17	85.523	83.333
GCA_023711345	82.156	83.376	80.373	84.419	84.531	86.092	87.1	84.623	81.203	85.976	90.135	89.381	87.805	87.214	91.69	81.043	85.523	83.333
GCA_023711365	82.156	83.376	80.373	84.419	84.531	86.092	87.1	84.623	81.203	85.976	90.135	89.381	87.805	87.214	91.69	81.043	85.523	83.333
GCA_023711385	82.156	83.376	80.373	84.419	84.531	86.092	87.1	84.623	81.203	85.976	90.135	89.381	87.642	87.214	91.69	81.17	85.523	83.333
GCA_023711405	82.156	83.376	80.373	84.419	84.531	86.092	87.1	84.623	81.203	85.976	90.135	89.381	87.805	87.214	91.69	81.043	85.523	83.333
GCA_023711445	82.156	83.376	80.373	84.419	84.531	86.092	87.1	84.623	81.203	85.976	90.135	89.381	87.805	87.214	91.69	81.043	85.523	83.333
GCA_023711485	82.156	83.376	80.373	84.419	84.531	86.092	87.1	84.623	81.203	85.976	90.135	89.381	87.805	87.214	91.69	81.043	85.523	83.333
GCA_023711505	82.156	83.12	80.373	84.419	84.531	86.092	87.1	84.623	81.203	85.976	90.135	89.381	87.724	87.11	91.69	81.17	85.523	83.333
GCA_023711525	82.156	83.12	80.373	84.419	84.531	86.092	87.1	84.623	81.203	85.976	90.135	89.381	87.805	87.11	91.69	81.043	85.523	83.333
GCA_023711565	82.156	83.376	80.373	84.419	84.531	86.092	87.1	84.623	81.203	85.976	90.135	89.381	87.805	87.214	91.69	81.043	85.523	83.333
GCA_023711605	82.156	83.376	80.373	84.419	84.531	86.092	87.1	84.623	81.203	85.976	90.135	89.381	87.805	87.214	91.69	81.043	85.523	83.333
GCA_023711645	82.156	83.376	80.373	84.419	84.531	86.092	87.1	84.623	81.203	85.976	90.135	89.381	87.805	87.214	91.69	81.043	85.523	83.333
GCA_023711685	82.156	83.376	80.373	84.419	84.531	86.092	87.1	84.623	81.203	85.976	90.135	89.381	87.805	87.214	91.69	81.043	85.523	83.333
GCA_023711725	82.156	83.376	80.373	84.419	84.531	86.092	87.1	84.623	81.203	85.976	90.135	89.381	87.805	87.214	91.69	81.043	85.523	83.333
GCA_023711765	82.156	83.376	80.373	84.419	84.531	86.092	87.1	84.623	81.203	85.976	90.135	89.381	87.805	87.214	91.69	81.043	85.523	83.333
GCA_023711805	82.156	83.376	80.373	84.419	84.531	86.092	87.1	84.623	81.203	85.976	90.135	89.381	87.805	87.214	91.69	81.043	85.523	83.333
GCA_023711825	82.156	83.376	80.373	84.419	84.531	86.092	87.1	84.623	81.203	85.976	90.135	89.381	87.724	87.214	91.69	81.17	85.523	83.333
GCA_023711845	82.156	83.376	80.373	84.419	84.531	86.092	87.1	84.623	81.203	85.976	90.135	89.381	87.805	87.214	91.69	81.043	85.523	83.333
GCA_023711885	82.156	83.376	80.373	84.419	84.531	86.092	87.1	84.623	81.203	85.976	90.135	89.381	87.805	87.214	91.69	81.043	85.523	83.333
GCA_023711905	82.156	83.376	80.373	84.419	84.531	86.092	87.1	84.623	81.203	85.976	90.135	89.381	87.724	87.214	91.69	81.17	85.523	83.333
GCA_023711925	82.156	83.376	80.373	84.419	84.531	86.092	87.1	84.623	81.203	85.976	90.135	89.381	87.805	87.214	91.69	81.043	85.523	83.333
GCA_023711965	82.156	83.376	80.373	84.419	84.531	86.092	87.1	84.623	81.203	85.976	90.135	89.381	87.805	87.214	91.69	81.043	85.523	83.333
GCA_023712005	82.156	83.376	80.373	84.419	84.531	86.092	87.1	84.623	81.203	85.976	90.135	89.381	87.805	87.214	91.69	81.043	85.523	83.333
GCA_023712045	82.156	83.376	80.373	84.419	84.531	86.092	87.1	84.623	81.203	85.976	90.135	89.381	87.805	87.214	91.69	81.043	85.523	83.333
GCA_023712065	82.156	83.376	80.373	84.419	84.531	86.092	87.1	84.623	81.203	85.976	90.135	89.381	87.642	87.214	91.69	81.17	85.523	83.333
GCA_023712085	82.156	83.376	80.373	84.419	84.531	86.092	87.1	84.623	81.203	85.976	90.135	89.381	87.805	87.214	91.69	81.043	85.523	83.333
GCA_023735475	82.156	83.376	80.373	84.419	84.531	86.092	87.1	84.623	81.203	85.976	90.135	89.381	87.805	87.214	91.736	81.043	85.523	83.333
GCA_023735495	82.156	83.376	80.373	84.419	84.531	86.092	87.1	84.623	81.203	85.976	90.135	89.381	87.805	87.214	91.736	81.043	85.523	83.333
GCA_023735515	82.156	83.376	80.373	84.419	84.531	86.092	87.1	84.623	81.203	85.976	90.135	89.381	87.805	87.214	91.69	81.043	85.523	83.333
GCA_023735535	82.156	83.376	80.373	84.419	84.531	86.092	87.1	84.623	81.203	85.976	90.135	89.381	87.805	87.214	91.69	81.043	85.523	83.333
GCA_023735615	82.156	83.376	80.373	84.419	84.531	86.092	87.1	84.623	81.203	85.976	90.135	89.381	87.805	87.214	91.69	81.043	85.523	83.333
GCA_023735695	82.156	83.376	80.373	84.419	84.531	86.092	87.1	84.623	81.203	85.976	90.135	89.381	87.805	87.214	91.69	81.043	85.523	83.333
GCA_023735715	82.156	83.376	80.373	84.419	84.531	86.092	87.1	84.623	81.203	85.976	90.135	89.381	87.805	87.214	91.69	81.043	85.523	83.333
GCA_023735735	82.156	83.12	80.373	84.419	84.531	86.092	87.1	84.623	81.203	85.976	90.135	89.381	87.805	87.11	91.69	81.043	85.523	83.333
GCA_023735755	82.156	83.376	80.373	84.419	84.531	86.092	87.1	84.623	81.203	85.976	90.135	89.381	87.805	87.214	91.69	81.043	85.523	83.333
GCA_023735775	82.156	83.376	80.373	84.419	84.531	86.092	87.1	84.623	81.203	85.976	90.135	89.381	87.805	87.214	91.69	81.043	85.523	83.333
GCA_023735795	82.156	83.376	80.373	84.419	84.531	86.092	87.1	84.623	81.203	85.976	90.135	89.381	87.805	87.214	91.69	81.043	85.523	83.333
GCA_023735815	82.156	83.376	80.373	84.419	84.531	86.092	87.1	84.623	81.203	85.976	90.135	89.381	87.805	87.214	91.69	81.043	85.523	83.333
GCA_023735835	82.156	83.376	80.373	84.419	84.531	86.092	87.1	84.623	81.203	85.976	90.135	89.381	87.805	87.214	91.69	81.043	85.523	83.333
GCA_023735855	82.156	83.376	80.373	84.419	84.531	86.092	87.1	84.623	81.203	85.976	90.135	89.381	87.805	87.214	91.69	81.043	85.523	83.333
GCA_023735875	82.156	83.376	80.373	84.419	84.531	86.092	87.1	84.623	81.203	85.976	90.135	89.381	87.805	87.214	91.69	81.043	85.523	83.333
GCA_023735895	82.156	83.376	80.373	84.419	84.531	86.092	87.1	84.623	81.203	85.976	90.135	89.381	87.805	87.214	91.69	81.043	85.523	83.333
GCA_023735915	82.156	83.376	80.373	84.419	84.531	86.092	87.1	84.623	81.203	85.976	90.135	89.381	87.805	87.214	91.69	81.043	85.523	83.333
GCA_023735935	82.156	83.376	80.373	84.419	84.531	86.092	87.1	84.623	81.203	85.976	90.135	89.381	87.805	87.214	91.69	81.043	85.523	83.333
GCA_023735955	82.156	83.376	80.373	84.419	84.531	86.092	87.1	84.623	81.203	85.976	90.135	89.381	87.805	87.214	91.69	81.043	85.523	83.333
GCA_023735975	82.156	83.376	80.373	84.419	84.531	86.092	87.1	84.623	81.203	85.976	90.135	89.381	87.805	87.214	91.69	81.043	85.523	83.333
GCA_023735995	82.156	83.376	80.373	84.419	84.531	86.092	87.1	84.623	81.203	85.976	90.135	89.381	87.805	87.214	91.69	81.043	85.523	83.333
GCA_023736015	82.156	83.376	80.373	84.419	84.531	86.092	87.1	84.623	81.203	85.976	90.135	89.381	87.805	87.214	91.69	81.043	85.523	83.333
GCA_023749035	82.156	83.376	80.263	84.419	84.531	86.092	87.1	84.623	81.203	85.976	90.135	89.381	87.805	87.214	91.736	81.043	85.523	83.333
GCA_023749505	82.156	83.376	80.373	84.419	84.531	86.092	87.1	84.623	81.203	85.976	90.135	89.381	87.724	87.214	91.736	81.043	85.523	83.333
GCA_023749525	82.156	83.376	80.373	84.419	84.531	86.092	87.1	84.623	81.203	85.976	90.135	89.381	87.724	87.214	91.736	81.043	85.523	83.333
GCA_023753905	82.156	83.376	80.373	84.419	84.531	86.092	87.1	84.623	81.203	85.976	90.135	89.381	87.805	87.214	91.736	81.043	85.523	83.333
GCA_023762255	82.156	83.376	80.373	84.419	84.531	86.092	87.1	84.623	81.203	85.976	90.135	89.381	87.805	87.214	91.736	81.043	85.523	83.333
GCA_023803735	82.156	83.376	80.373	84.419	84.531	86.092	87.1	84.623	81.203	85.976	90.135	89.381	87.805	87.11	91.736	81.043	85.523	83.333
GCA_024138915	82.156	83.376	80.373	84.419	84.531	86.092	87.1	84.623	81.203	85.976	90.135	89.381	87.805	87.214	91.736	81.043	85.523	83.333
GCA_900074995	82.156	83.376	80.373	84.419	84.531	86.092	87.1	84.623	81.203	85.976	90.135	89.354	87.805	87.214	91.736	81.043	85.523	83.333
GCA_900075055	82.156	83.376	80.373	84.419	84.531	86.092	87.1	84.623	81.203	85.976	90.135	89.381	87.805	87.214	91.736	81.043	85.523	83.333
GCA_900075115	82.156	83.376	80.373	84.419	84.531	86.092	87.1	84.623	81.203	85.976	90.135	89.381	87.805	87.214	91.736	81.043	85.523	83.209
GCA_900075155	82.156	83.376	80.373	84.419	84.531	86.092	87.1	84.623	81.203	85.976	90.135	89.381	87.805	87.214	91.736	81.043	85.523	83.333
GCA_900075275	82.156	83.12	80.373	84.419	84.531	86.092	87.1	84.623	81.203	85.976	90.135	89.381	87.805	87.214	91.736	81.043	85.523	83.333
GCA_900075325	82.156	83.376	80.373	84.419	84.531	86.092	87.1	84.524	81.203	85.976	90.135	89.381	87.805	87.11	91.736	81.043	85.523	83.333
GCA_900075675	82.156	83.376	80.373	84.419	84.531	86.092	87.1	84.623	81.203	85.976	90.135	89.381	87.805	87.214	91.736	81.043	85.523	83.333
GCA_900075835	82.156	83.376	80.373	84.419	84.531	86.092	87.1	84.623	81.203	85.976	90.135	89.381	87.805	87.214	91.736	81.043	85.523	83.333
GCA_900075865	82.156	83.376	80.373	84.419	84.531	86.092	87.1	84.623	81.203	85.976	90.135	89.381	87.805	87.214	91.736	81.043	85.523	83.333
GCA_900076195	82.156	83.376	80.373	84.419	84.531	86.092	87.1	84.623	81.203	85.976	90.135	89.381	87.805	87.214	91.736	81.043	85.523	83.333
GCA_900076215	82.156	83.376	80.373	84.419	84.531	86.092	87.1	84.623	81.203	85.976	90.135	89.381	87.805	87.214	91.736	81.043	85.523	83.333
GCA_900076315	82.156	83.376	80.373	84.419	84.531	86.092	87.1	84.623	81.203	85.976	90.135	89.381	87.805	87.214	91.736	81.043	85.523	83.333
GCA_900076705	82.156	83.376	80.373	84.419	84.531	86.092	87.1	84.623	80.827	85.976	90.135	89.381	87.805	87.214	91.736	81.043	85.523	83.333
GCA_900076965	82.156	83.376	80.373	84.419	84.531	86.092	87.1	84.623	81.203	85.976	90.135	89.381	87.805	87.214	91.736	81.043	85.523	83.333
GCA_900077255	82.156	83.376	80.373	84.419	84.531	86.092	87.1	84.623	81.203	85.976	90.135	89.354	87.805	87.214	91.736	81.043	85.523	83.333
GCA_900077415	82.156	83.376	80.373	84.419	84.531	86.092	87.1	84.623	81.203	85.976	90.135	89.381	87.805	87.214	91.736	81.043	85.523	83.333
GCA_900077435	82.156	83.376	80.373	84.419	84.531	86.092	87.1	84.623	81.203	85.976	90.135	89.381	87.805	87.214	91.736	81.043	85.523	83.333
GCA_900077445	82.156	83.376	80.373	84.419	84.531	86.092	87.1	84.623	81.203	85.976	90.135	89.381	87.805	87.214	91.736	81.043	85.523	83.333
GCA_900077595	82.156	83.376	80.373	84.419	84.531	86.092	87.1	84.623	81.203	85.976	90.135	89.381	87.805	87.214	91.736	81.043	85.523	83.333

GCA_900077825	82.156	83.376	80.373	84.419	84.531	86.092	87.1	84.623	81.203	85.976	90.135	89.381	87.805	87.214	91.736	81.043	85.523	83.333
GCA_900077865	82.156	83.376	80.373	84.419	84.531	86.092	87.1	84.623	81.203	85.976	90.135	89.381	87.805	87.214	91.736	81.043	85.523	83.333
GCA_900077915	82.156	83.376	80.373	84.419	84.531	86.092	87.1	84.623	81.203	85.976	90.135	89.381	87.805	87.214	91.736	81.043	85.523	83.333
GCA_900078035	82.156	83.376	80.373	84.419	84.531	86.092	87.1	84.623	81.203	85.976	90.135	89.381	87.805	87.214	91.736	81.043	85.523	83.333
GCA_900078095	82.156	83.376	80.373	84.419	84.531	86.092	87.1	84.623	81.203	85.976	90.135	89.381	87.805	87.214	91.736	81.043	85.523	83.333
GCA_900557965	82.156	83.376	80.373	84.419	84.531	86.092	87.1	84.623	81.203	85.976	90.135	89.381	87.805	87.214	91.736	81.043	85.523	83.333
GCA_900557985	82.156	83.376	80.373	84.419	84.531	86.092	87.1	84.623	81.203	85.976	90.135	89.381	87.805	87.214	91.736	81.043	85.523	83.333
GCA_900558115	82.156	83.376	80.373	84.419	84.531	86.092	87.1	84.623	81.203	85.976	90.135	89.381	87.805	87.214	91.736	81.043	85.523	83.333
GCA_900558225	82.156	83.376	80.373	84.419	84.531	86.092	87.1	84.623	81.203	85.976	90.135	89.381	87.805	87.214	91.736	81.043	85.523	83.333
GCA_900558345	82.156	83.376	80.373	84.419	84.531	86.092	87.1	84.623	81.203	85.976	90.135	89.381	87.805	87.214	91.736	81.043	85.523	83.333
GCA_900558425	82.156	83.376	80.373	84.419	84.531	86.092	87.1	84.623	81.203	85.976	90.135	89.381	87.805	87.214	91.69	81.043	85.523	83.333
GCA_900558435	82.156	83.376	80.373	84.419	84.531	86.092	87.1	84.623	81.203	85.976	90.135	89.381	87.805	87.214	91.736	81.043	85.523	83.333
GCA_900558555	82.156	83.376	80.373	84.419	84.531	86.092	87.1	84.623	81.203	85.976	90.135	89.381	87.805	87.214	91.736	81.043	85.523	83.333
GCA_900558655	82.156	83.376	80.373	84.419	84.531	86.092	87.1	84.623	81.203	85.976	90.135	89.409	87.805	87.214	91.736	81.043	85.523	83.333
GCA_900558985	82.156	83.376	80.373	84.419	84.531	86.092	87.1	84.623	81.203	85.976	90.135	89.381	87.805	87.214	91.736	81.043	85.523	83.333
GCA_900559015	82.156	83.376	80.373	84.419	84.531	86.092	87.1	84.623	81.203	85.976	90.135	89.381	87.805	87.214	91.736	81.043	85.523	83.333
GCA_900559025	82.156	83.376	80.373	84.291	84.531	86.092	87.1	84.623	81.203	85.976	90.135	89.381	87.805	87.214	91.736	81.043	85.523	83.333
GCA_900559045	82.156	83.376	80.373	84.419	84.531	86.092	87.1	84.623	81.203	85.976	90.135	89.381	87.805	87.214	91.736	81.043	85.523	83.333
GCA_903936115	82.156	83.376	80.373	84.419	84.531	86.092	87.1	84.623	81.203	85.976	90.135	89.381	87.805	87.214	91.736	81.043	85.523	83.333
GCA_903936205	82.156	83.376	80.373	84.419	84.531	86.092	87.1	84.623	81.203	85.976	90.135	89.381	87.805	87.214	91.736	81.043	85.523	83.333
GCA_903936215	82.156	83.376	80.373	84.419	84.531	86.092	87.1	84.623	81.203	85.976	90.135	89.381	87.805	87.214	91.736	81.043	85.523	83.333
GCA_903936225	82.156	83.376	80.373	84.419	84.531	86.092	87.1	84.623	81.203	85.976	90.135	89.381	87.805	87.214	91.736	81.043	85.523	83.333
GCA_905232475	82.156	83.12	80.373	84.419	84.531	86.092	87.1	84.623	81.203	85.976	90.135	89.381	87.805	87.214	91.736	81.043	85.523	83.333
GCA_905232485	82.156	83.12	80.373	84.419	84.531	86.092	87.1	84.623	81.203	85.976	90.135	89.381	87.805	87.214	91.736	81.043	85.523	83.333
GCA_905232495	82.156	83.12	80.373	84.419	84.531	86.092	87.1	84.623	81.203	85.976	90.135	89.381	87.805	87.214	91.736	81.043	85.523	83.333
GCA_905330285	82.156	83.376	80.373	84.419	84.531	86.092	87.1	84.623	81.203	85.976	90.135	89.381	87.805	87.214	91.736	81.043	85.523	83.333
GCA_905330795	82.156	83.12	80.373	84.419	84.531	86.092	87.1	84.623	81.203	85.976	90.135	89.381	87.805	87.214	91.736	81.043	85.523	83.333
GCF_019537235	82.156	83.376	80.373	84.419	84.531	86.092	87.1	84.623	81.203	0	90.135	89.381	87.805	87.214	91.736	81.043	85.523	83.333
GCF_019537335	82.156	83.376	80.373	84.419	84.531	86.092	87.1	84.623	81.203	85.976	90.135	89.381	87.805	87.214	91.736	81.043	85.523	83.333
